# Correlation between subjective and objective hearing tests after unilateral and bilateral cochlear implantation

**DOI:** 10.1186/s12901-017-0043-y

**Published:** 2017-11-28

**Authors:** Geerte G. J. Ramakers, Yvette E. Smulders, Alice van Zon, Gijsbert A. Van Zanten, Wilko Grolman, Inge Stegeman

**Affiliations:** 1 Department of Otorhinolaryngology, Head and Neck Surgery, University Medical Center Utrecht, Utrecht University, P.O. Box 85500, 3508 GA Utrecht, The Netherlands; 2 Brain Center Rudolf Magnus, University Medical Center Utrecht, Universiteitsweg 100, 3584 CG Utrecht, The Netherlands

**Keywords:** Cochlear implantation, Unilateral, Bilateral, Subjective, Objective, Hearing tests, Correlation

## Abstract

**Background:**

There are many methods for assessing hearing performance after cochlear implantation. Standard evaluations often encompass objective hearing tests only, while patients’ subjective experiences gain importance in today’s healthcare. The aim of the current study was to analyze the correlation between subjective (self-reported questionnaires) and objective (speech perception and localization) hearing test results in adult cochlear implant (CI) users. Secondary, the correlation between subjective and objective hearing tests was compared between bilateral and unilateral CI patients.

**Methods:**

Data for this study were prospectively collected as part of a multicentre randomized controlled trial. Thirty-eight postlingually deafened adult patients were randomly allocated to receive either unilateral (*n* = 19) or bilateral (*n* = 19) cochlear implantation. We used data gathered after one year of follow-up. We studied the correlation between objectively measured speech perception and localization skills on the one hand and related domains of the Speech, Spatial and Qualities of Hearing Scale (SSQ) and Nijmegen Cochlear Implant Questionnaire (NCIQ) on the other hand. We also compared these correlations between unilateral and bilateral CI users.

**Results:**

We found significant weak to moderate negative correlations between the subjective test results (speech domain of the SSQ and the advanced speech perception domain of the NCIQ) and the related objective speech perception in noise test results (*r* = −0.33 to −0.48). A significant moderate correlation was found between the subjective test results (spatial domain of the SSQ) and the related objective localization test results (*r* = 0.59). The correlations in the group of bilateral CI patients (*r* = −0.28 to −0.54) did not differ significantly from the correlations in the group of unilateral CI patients (*r* = 0.15 to −0.40).

**Conclusions:**

Current objective tests do not fully reflect subjective everyday listening situations. This study elucidates the importance and necessity of questionnaires in the evaluation of cochlear implantation. Therefore, it is advised to evaluate both objective and subjective tests in CI patients on a regular basis.

**Trial registration:**

This trial was registered on March 11, 2009 in the Dutch Trial Register. Trial registration number: NTR1722.

## Background

Cochlear implantation is a successful treatment for severe to profound sensorineural hearing loss. Although unilateral cochlear implantation still is the standard treatment in most countries, an increasing amount of patients worldwide is being implanted bilaterally in order to improve (spatial) hearing skills and speech understanding in noise [1, 2].

The eligibility criteria for cochlear implantation are constantly changing and the quality and possibilities of cochlear implants (CIs) are growing [3]. In this world of new developments, assessing hearing performance after cochlear implantation is vital. There are various methods to do this. In many CI centres, evaluations encompass objective hearing tests only. Clinically applied speech perception and localization tests are robust and reliable, but time-consuming and it is questionable if these test conditions fully represent everyday listening situations. Subjective tests (self-reported questionnaires) are easy to administer and a large set of data can be gathered in a short period of time. Also, in today’s healthcare, a patients’ subjective experiences gain importance [4, 5]. For example, when the cost-effectiveness of a treatment is analyzed, health related quality of life questionnaires are often used to measure the effectiveness [6, 7]. However, questions can be misinterpreted and missing values easily occur when patients do not fill out (parts of) the questionnaires.

Literature has shown that there are often discrepancies between subjective and objective hearing test results [8–13]. Previous studies were mainly about the correlation between subjective and objective speech perception tests. The amount of literature on correlations between subjective and objective localization tests is limited [10].

There is an ongoing global discussion on whether or not bilateral cochlear implantation should be standard care for bilateral deafness [1, 2]. The current literature on correlations between subjective and objective tests however, only includes unilateral and bimodal CI users. Correlations between tests might be different for unilateral and bilateral CI users, due to differences in test sensitivity or differences in indicating their own performance. Therefore, the latter is worth investigating.

The current study is a subanalysis of a previous published study on the comparison of bilateral and unilateral cochlear implantation in adult patients with bilateral postlingual deafness [14]. One year after implantation, bilaterally implanted patients performed significantly better on part of the subjective (Speech, Spatial and Qualities of Hearing Scale (SSQ) and the visual analogue scale (VAS) on hearing) and objective (speech perception in noise when noise came from different directions and localization of sounds) tests [14].

The first objective of the current study was to investigate the correlations between subjective and objective speech perception and localization tests in adult CI patients. Secondary, the correlations between subjective and objective speech perception and localization tests were compared between bilateral and unilateral CI patients.

## Methods

### Study design and participants

The current study will present the results of a secondary analysis of data collected as part of a multicentre randomized controlled trial on the benefits of simultaneous bilateral cochlear implantation compared to unilateral cochlear implantation in adults with severe to profound bilateral postlingual sensorineural hearing loss [14]. Between December 2009 and September 2012, 38 adult patients were included in this study. After giving informed consent, patients were randomly allocated to receive cochlear implants bilaterally or unilaterally. All patients were implanted with Advanced Bionics HiRes90K (Advanced Bionics, Sylmar, California) CIs and used Harmony processors.

In this paper, we will present the correlation between subjective and objective hearing tests measured one year after implantation. Detailed descriptions of the study methods and the main study results have been reported previously [14, 15].

### Subjective hearing outcomes

Subjective benefits in everyday listening situations were assessed with the following questionnaires:

1. Speech, Spatial and Qualities of Hearing Scale (SSQ). This questionnaire consists of three domains of questions. Participants were asked to rate their hearing capabilities on a 0–100 scale (0 = not capable at all, 100 = perfectly capable). The SSQ1 comprises questions on speech understanding in silence, in background noise, in resonating environments and on the telephone. The SSQ2 comprises questions on spatial hearing; identifying directions of sounds and distance approximation, and the SSQ3 encompasses questions on the quality of hearing [16]. The final subdomain score is computed by the mean of all items on that subdomain, resulting in a range of scores from 0 to 100. A higher score reflects a greater ability [16].
2. Nijmegen Cochlear Implant Questionnaire (NCIQ). This questionnaire contains six subdomains of hearing that are rated categorically (1–5 (never-always) and “not applicable”). The subdomains are 1. Basic sound perception, 2. Advanced sound perception (in difficult daily listening situations or background noise), 3. Speech production, 4. Self-esteem, 5. Activity limitations, 6. Social interaction [17]. The answer categories must first be transformed (1 = 0, 2 = 25, 3 = 50, 4 = 75 and 5=100). Afterwards, the final subdomain score is computed by adding together all the item scores and dividing by the number of completed items, resulting in a range of scores from 0 to 100. A higher score reflects a greater ability [17].

### Objective hearing outcomes

Speech perception in noise and sound localization tests were conducted with the Dutch version of the AB-York crescent of sound. The test battery included the Utrecht Sentence Test with Adaptive Randomized Roving levels (U-STARR), the speech-intelligibility test with spatially separated sources (SISSS), and a sound localization test [15].

1. With the U-STARR, sentences were presented in noise, both coming from straight ahead. The sentences were presented at 65, 70 or 75 dB SPL (randomly selected), in noise with an adaptive level. The outcome was the signal-to-noise ratio (SNR) average of the last sixteen sentences, which is the speech reception threshold in noise (SRTn) [15].
2. For the SISSS, the same procedure was used as for the U-STARR. The only difference was that the sentences were presented from 60° to the left (−60° azimuth) or to the right (+60° azimuth) of the subject and the noise was presented from 60° at the opposite side [15].

A SRTn of 30 dB was considered relative silence and therefore, 30 dB was used as cutoff value on the U-STARR and SISSS.

3. For the sound localization test, a phrase ‘Hello what’s this?’ was randomly presented from loudspeakers at 0°, ±15°, ±30° and ±60° angles, about 30 times per condition. Again, the phrase was randomly presented at 60, 65, or 70 dB SPL. The result of this test was the percentage of correct responses [15]. In the current article, the average of all three conditions was used as the localization score.

In the unilateral group, patients were encouraged to use a contralateral hearing aid (HA). The scores on the objective tests in their daily hearing situation (only CI or CI + HA) were used for the analyses. When sounds come from different directions, patients usually have a “best performing situation” and a “worst performing situation”. A patient’s “best performing situation” occurs when sound is presented to the best hearing ear and noise to the worst hearing ear. In the unilateral group, the best hearing ear is the implanted ear. In the bilateral group, patients usually also have one ear with which they hear (slightly) better than with the other. We defined the “best performing situation” and “worst performing situation” for each patient [14].

### Statistical analysis

None of the subjective and objective test results were normally distributed. Therefore, medians, interquartile ranges (IQR) and non-parametric tests were used.

In order to get insight in the relation between the subjective and objective tests, scatter plots of individual patient scores were created with the subjective test score on the x-axis and the related objective test score on the y-axis.

We used Spearman correlation tests to quantify the relationship between subjective and objective test results. We studied the relation between the U-STARR and SISSS scores (objective) and the first domain of the SSQ (SSQ1) and the advanced sound perception domain of the NCIQ (subjective). These tests all represent advanced sound perception skills. The second domain of the SSQ (SSQ2) contains questions on sound localization, thus, we studied the relation between the SSQ2 and the objective sound localization test.

The correlations between subjective and objective tests were analyzed for the whole study group (*n* = 38), and for the bilateral and unilateral CI patients separately. We used the Fisher’s z transformation to analyze if there was a statistical significant difference between the correlations in the bilateral and unilateral CI group.

A correlation of <0.19 is considered very weak, 0.20–0.39 weak, 0.40–0.59 moderate, 0.60–0.79 strong, >0.80 very strong (for positive as well as negative values) [18]. For the speech in noise tests (U-STARR and SISSS), a low result indicates good performance, while for the localization tests and subjective tests, a high score indicates good performance. For this reason, when speech in noise results are compared with subjective outcomes, correlations are often negative. All data were analyzed using SPSS 22.0. The critical significance levels of the *p*-values were adjusted for multiple comparisons using the Benjamini-Hochberg false discovery rate method [19].

## Results

Details of the study population are presented in Table 1. Fifteen patients in the bilateral CI group used HAs before implantation, compared to 19 patients in the unilateral group (*p:* 0.04) [14]. All other baseline characteristics did not differ significantly. One year after cochlear implantation, 14 out of 19 patients in the unilateral group still used a contralateral HA.

**Table 1:** Baseline characteristics

	Bilateral	Unilateral
Number of participants	19	19
Male number (%)	8 (42)	11 (58)
Age at inclusion years, median [IQR]	52 [36–63]	54 [43–64]
Duration severe hearing loss right ear years, medians [IQR]	16 [11–25]	17 [9–33]
Duration severe hearing loss left ear years, median [IQR]	16 [11–25]	18 [9–35]
PTA right ear decibels, median [IQR]	106 [89–119]	106 [94–111]
PTA left ear decibels, median [IQR]	108 [89–120]	108 [93–114]
Hearing aid use before CI number/total	19/19	15/19

PTA: pure tone average at 1, 2 and 4 kHz

### Correlation between subjective and objective speech perception tests

**Fig. 1:**
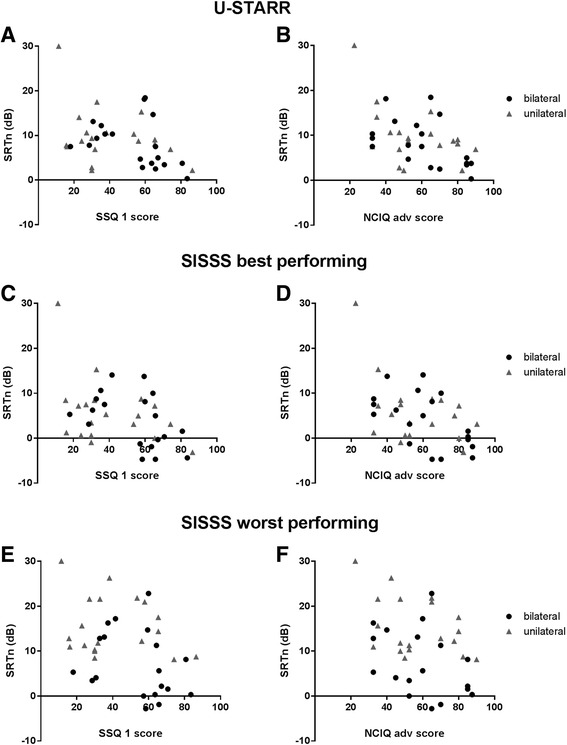
Correlation between subjective and objective speech perception results. Legend: Scatter plots of individual subjective and objective speech perception results. The correlation between the speech domain of the SSQ and the U-STARR (**a**). The correlation between the advanced speech perception domain of the NCIQ and the U-STARR (**b**). The correlation between the speech domain of the SSQ and the SISSS in the best (**c**) and worst (**e**) performing situation. The correlation between the advanced speech perception domain of the NCIQ and the SISSS in the best (**d**) and worst (**f**) performing situation

Figure 1 presents scatter plots of the individual patient scores on the subjective (SSQ1 and the advanced speech perception domain of the NCIQ) and objective speech perception tests (U-STARR and SISSS). The correlations between all these subjective and objective speech perception tests were weak to moderate, but significant (Table 2). The weakest correlation was found for the ‘SSQ1’ and ‘SISSS worst performing situation’ (*r* = −0.33, *p* = 0.046) and the strongest correlation for the ‘NCIQ advanced speech perception’ and ‘SISSS best performing situation’ (*r* = −0.48, *p* = 0.002). The ‘NCIQ advanced speech perception domain’ correlated better with the different objective speech perception tests (*r* between −0.39 and −0.48 corresponding moderate correlations) than the SSQ1 (*r* between −0.33 and −0.39, corresponding with weak correlations).

**Table 2:** Correlation between subjective and objective hearing tests. Results for all cochlear implant patients (*n* = 38)

	U-STARR		Corrected significance level^a^
	Spearman r	*p*-value	
SSQ 1 (Speech in silence and noise)	−0.36	0.028	0.0429
NCIQ advanced speech perception	−0.47	0.003	0.0214
	SISSS Best performing situation		
	Spearman r	*p*-value^a^	
SSQ 1 (Speech in silence and noise)	−0.39	0.016	0.0286
NCIQ advanced speech perception	−0.48	0.002	0.0143
	SISSS Worst performing situation		
	Spearman r	*p*-value^a^	
SSQ 1 (Speech in silence and noise)	−0.33	0.046	0.05
NCIQ advanced speech perception	−0.39	0.016	0.0357
	Localization		
	Spearman r	*p*-value^a^	
SSQ 2 (Spatial hearing)	0.59	0.0001	0.0071

r: <0.19 = very weak, r 0.20–0.39 = weak, r 0.40–0.59 = moderate, r 0.60–0.79 = strong, *r* > 0.80 = very strong. U-STARR = Utrecht- Sentence Test with Adaptive Randomised Roving levels, SSQ = Speech, Spatial and Qualities hearing scale. NCIQ = Nijmegen CI Questionnaire, SISSS = speech-intelligibility test with spatially separated sources (SISSS)

^a^The for multiple testing corrected significance level with the Benjamini-Hochberg false discovery rate method

### Correlation between subjective and objective localization tests

**Fig. 2:**
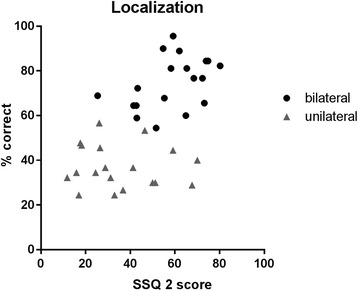
Correlation between subjective and objective sound localization results. Legend: Scatter plot of the spatial domain of the SSQ and the objective localization test

Figure 2 presents a scatter plot for the individual patient scores on the subjective (SSQ2) and objective localization test. A significant moderate correlation was found between the SSQ2 and localization test (*r =* 0.59, *p* = 0.0001) (lower part of Table 2).

When we corrected for multiple testing using the Benjamini-Hochberg false discovery rate method, all *p*-values of the correlation coefficients were lower than the for multiple testing corrected significance level, resulting in all significant correlations (Table 2).

### Comparison of correlations between bilateral and unilateral CI patients

As presented in Table 3, the correlations between all subjective and objective hearing tests ranged between −0.28 and −0.55 (weak to moderate) in the bilateral CI group, compared to a range of −0.15 to −0.43 (very weak to moderate) in the unilateral CI group. The correlation coefficients in the bilateral group did not differ significantly from the correlation coefficients in the unilateral group, after correction for multiple testing using the Benjamini-Hochberg false discovery rate method (Table 3).

**Table 3:** Correlation between subjective and objective hearing tests. Results for bilateral (*n* = 19) and unilateral patients (*n* = 19) separately

	U-STARR				*p*-value comparison correlation^a^
	Bilateral		Unilateral		
	Spearman r	*p*-value^a^	Spearman r	*p*-value^a^	
SSQ 1 (Speech in silence and noise)	−0.50	0.031	−0.21	0.379	0.342
NCIQ advanced sound perception	−0.55	0.014	−0.43	0.067	0.653
	SISSS best performing situation				
	Bilateral		Unilateral		
	Spearman r	*p*-value^a^	Spearman r	*p*-value^a^	
SSQ 1 (Speech in silence and noise)	−0.44	0.057	−0.29	0.230	0.624
NCIQ advanced sound perception	−0.54	0.016	−0.38	0.109	0.562
	SISSS worst performing situation				
	Bilateral		Unilateral		
	Spearman r	*p*-value^a^	Spearman r	*p*-value^a^	
SSQ 1 (Speech in silence and noise)	−0.28	0.247	−0.15	0.544	0.697
NCIQ advanced sound perception	−0.43	0.067	−0.38	0.110	0.865
	Localization				
	Bilateral		Unilateral		
	Spearman r	*p*-value^a^	Spearman r	*p*-value^a^	
SSQ 2 (Spatial hearing)	0.47	0.042	−0.22	0.929	0.038

r: <0.19 = very weak, r 0.20–0.39 = weak, r 0.40–0.59 = moderate, r 0.60–0.79 = strong, r > 0.80 = very strong. U-STARR = Utrecht- Sentence Test with Adaptive Randomised Roving levels, SSQ = Speech, Spatial and Qualities hearing scale. NCIQ = Nijmegen CI Questionnaire, SISSS = speech-intelligibility test with spatially separated sources (SISSS)

^a^After correction for multiple testing with the Benjamini-Hochberg false discovery rate procedure, none of the test results yielded significant results

## Discussion

### Key findings

In this study, we found significant correlations between subjective and objective hearing test results in adult CI users. The strongest correlation was found between the spatial domain of the SSQ and the objective localization test (*r* = 0.59, a moderate correlation). The other correlations, between subjective and objective speech perception in noise test results, were weak to moderate. There could be several reasons for the lack of strong correlations between subjective and objective results. Perhaps the questionnaires and objective tests do not represent the same hearing skills. Another reason could be that the patients’ views of their own hearing skills did not match their actual hearing capabilities. Therefore, it seems important to evaluate both subjectively and objectively measured hearing skills after cochlear implantation.

When we compared the outcomes of the unilateral and bilateral CI group, all correlations in the bilateral CI group were stronger than in the unilateral group, although none of the correlations differed statistically significant from each other. We cannot rule out that the latter is the result of the small sample size: 19 patients in each group.

### Comparison with the literature

A recently published meta-analysis reviewed the correlation between different types of (subjective) hearing-specific and CI-specific questionnaires and (objective) speech perception scores in CI patients [8]. Thirteen studies were included. These studies showed low correlations between hearing-specific and CI-specific questionnaires on the one hand and objective speech perception scores on the other hand [8]. The pooled correlation between CI-specific questionnaire scores (for example NCIQ) and speech perception in noise was weak (*r* = 0.26, *p*:0.0064). Other studies, not included in the meta-analysis, also found predominantly weak to moderate correlations between subjective and objective speech perception tests [9–13]. In a study of Hirschfelder et al. subjective and objective hearing tests were compared in 56 unilateral CI users [11]. They found significant weak to moderate (*r* = 0.28–0.56) correlations between the NCIQ total score, the NCIQ advanced sound perception, the NCIQ speech production domains and both objective speech perception tests (Freiburger monosyllable test in quiet and Hochmair, Schulz, Mozer (HSM) sentence test in noise). Damen et al. studied 69 postlingually deafened adult patients (59 unilaterally implanted and 10 non-implanted) and found significant correlations between the NCIQ total score and two Dutch standardized speech perception tests in quiet (the Antwerp-Nijmegen syllable (*r* = 0.48) and the NVA phoneme test (*r* = 0.32)) [9]. In a study of Brendel et al. the Everyday Listening Questionnaire (ELQ) 2 was significantly correlated to objective speech perception tests (Monosyllables, HSM in quiet and HSM in noise), but the strength of the correlations was not mentioned [12]. To date, only one study included objective spatial hearing tests [10]. Heo et al. reviewed the correlation between all domains of the SSQ and objective speech perception and localization tests in 14 unilateral CI recipients with a contralateral HA [10]. The spatial domain of the SSQ was significantly correlated with the environmental sound localization (*r* = 0.57) and perception (*r* = 0.55) scores. The quality domain was significantly correlated with all perception scores (*r* = 0.54–0.66) [10]. To our knowledge, there is no previous literature on the differences in correlations between bilateral and unilateral CI patients.

A drawback of some of the previous studies is the lack of clear hypotheses. That has resulted in the presentation of multiple random correlations between objective test scores and questionnaire scores without clear clinical relevance. Also, the authors did not correct for multiple testing. Nevertheless, our findings are in agreement with the previous literature, and our study methodologically fills the gaps of previously mentioned studies. We chose to study only clinically relevant relations by combining (parts of the) subjective tests with corresponding objective tests. To minimise the chance of finding incidental results we corrected for multiple testing. Another strength of our study is the use of prospectively collected data. All participants had completed the questionnaires one year after implantation and had performed the objective tests within the same week. None of the participants were lost to follow-up and we did not have any missing data. Also, to our knowledge our study is the first to investigate correlations between subjective and objective test results in bilateral CI patients. A weakness of the study is the small sample size. This might be the reason why we found some insignificant results after correcting for multiple testing.

## Conclusion

In this study, correlations between subjective and objective speech perception and spatial hearing tests were weak to moderate, but significant, in adult CI patients. The correlation between subjective and objective hearing tests seemed not different for bilateral compared to unilateral CI patients. This study elucidates the importance and necessity of questionnaires in the evaluation of cochlear implantation. Also it shows that patients may experience their own hearing performance differently than objective tests would suggest. Therefore, it is advised to use both objective and subjective tests in cochlear implant patients on a regular basis.

## Abbreviations

CI: Cochlear implant
dB SPL: Decibel, sound pressure level
ELQ: Everyday listening questionnaire
HA: Hearing aid
HSM: Hochmair, Schulz, Mozer
IQR: Interquartile range
NCIQ: Nijmegen cochlear implant questionnaire
NVA: Nederlandse Vereniging voor Audiologie (Dutch association for Audiology)
SISSS: The speech-intelligibility test with spatially separated sources
SNR: Signal-to-noise ratio
SRTn: Speech reception threshold in noise
SSQ: Speech, spatial and qualities of hearing scale
U-STARR: Utrecht Sentence Test with Adaptive Randomized Roving levels
VAS: Visual analogue scale

## References

[1] van Schoonhoven J, Sparreboom M, van Zanten BG, et al. the effectiveness of bilateral Cochlear implants for severe-to-profound deafness in Adults: a systematic review. Otol Neurotol. 2013;34(2):190–8. doi:10.1097/MAO.0b013e3181e3d62c. 10.1097/mao.0b013e318278506d 23444466

[2] Basura GJ, Eapen R, C a B (2009). Bilateral cochlear implantation: current concepts, indications, and results. Laryngoscope.

[3] Hwang CF, Chen Y, Lin HC, Narayanan P, SH O, Truy E (2015). Cochlear implant and its related science. Biomed Res Int.

[4] Deshpande PR, Rajan S, Lakshmi Sudeepthi B. Patient-reported outcomes : A new era in clinical research. 2011;2(4). doi:10.4103/2229-3485.86879. 10.4103/2229-3485.86879 PMC3227331 22145124

[5] Capretta NR, Moberly AC. Does quality of life depend on speech recognition performance for adult Cochlear implant Users ? March. 2016:699–706. doi:10.1002/lary.25525. 10.1002/lary.25525 26256441

[6] Ramakers GGJ, Smulders YE. Van Zon A, et al. Agreement between health utility instruments in cochlear implantation. Clin Otolaryngol. 2016;41(6):737–43. doi:10.1111/coa.12626. 10.1111/coa.12626 26868059

[7] Torrance GW. Utility approach to measuring health-related quality of. life. 1987;40(6) 10.1016/0021-9681(87)90019-1 3298297

[8] Mcrackan TR, Bauschard M, Hatch JL, et al. Meta-analysis of Quality-of-Life Improvement After Cochlear Implantation and Associations With Speech Recognition Abilities. 2017:1-9. doi:10.1002/lary.26738. 10.1002/lary.26738 PMC5776066 28731538

[9] Damen GWJA, Beynon AJ, Krabbe PFM, Mulder JJS, Mylanus EAM. Cochlear implantation and quality of life in postlingually deaf adults: long-term follow-up. Otolaryngol - Head Neck Surg. 2007;136(4):597–604. doi:10.1016/j.otohns.2006.11.044. 10.1016/j.otohns.2006.11.044 17418258

[10] Heo J-H, Lee J-H, Lee W-S (2013). Bimodal benefits on objective and subjective outcomes for adult cochlear implant users. Korean J Audiol.

[11] Hirschfelder A, Gräbel S, Olze H (2008). The impact of cochlear implantation on quality of life: the role of audiologic performance and variables. Otolaryngol - Head Neck Surg.

[12] Brendel M, Frohne-Buechner C, Lesinski-Schiedat A, Lenarz T, Buechner A (2014). Everyday listening questionnaire: correlation between subjective hearing and objective performance. Cochlear Implants Int.

[13] Moberly AC, Harris MS, Boyce L, et al. Relating Quality of Life to Outcomes and Predictors in Adult Cochlear Implant Users : Are We Measuring the Right Things ? 2017:1-8. doi:10.1002/lary.26791. 10.1002/lary.26791 PMC6192249 28776711

[14] Smulders YE, van Zon A, Stegeman I, et al. Comparison of bilateral and unilateral Cochlear implantation in adults: a randomized clinical trial. JAMA Otolaryngol Head Neck Surg. 2016:1–8. doi:10.1001/jamaoto.2015.3305. 10.1001/jamaoto.2015.3305 26796630

[15] Smulders YE, Rinia AB, Pourier VEC (2015). Validation of the U-STARR with the AB-York crescent of sound, a new instrument to evaluate speech intelligibility in noise and spatial hearing skills. Audiol Neurotol Extra.

[16] Gatehouse S, Noble W (2004). The speech, spatial and qualities of hearing scale (SSQ). Int J Audiol.

[17] Hinderink JB, Krabbe PF, Van Den Broek P (2000). Development and application of a health-related quality-of-life instrument for adults with cochlear implants: the Nijmegen cochlear implant questionnaire. Otolaryngol Head Neck Surg.

[18] Website BMJ. http://www.bmj.com/about-bmj/resources-readers/publications/statistics-square-one/11-correlation-and-regression. Accessed 25 Sept 2017.

[19] Benajmini Y, Hochberg Y (1995). Controlling the false discovery Rate : a practical and powerful approach to multiple testing author ( s ): Yoav Benjamini and Yosef Hochberg Source: Journal of the Royal Statistical Society. Series B (methodological), Vol. 57, no. 1 published by. J R Stat Soc B.

